# Designing a framework for primary health care research in Canada: a scoping literature review

**DOI:** 10.1186/s12875-018-0839-x

**Published:** 2018-08-29

**Authors:** Stephanie Montesanti, Ardene Robinson-Vollman, Lee A. Green

**Affiliations:** 1 School of Public Health, University of Alberta, Edmonton, Canada; 2 Department of Community Health Sciences, University of Calgary, Calgary, Canada; 3 Department of Family Medicine and Dentistry, University of Alberta, Edmonton, Canada

**Keywords:** Primary health care, Research priorities, Research transfer, Canada

## Abstract

**Background:**

Despite significant investments to improve primary health care (PHC) delivery in Canada, provincial health care systems remain fragmented and uncoordinated. Canada’s commitment to strengthening PHC should be driven by robust research and evaluation that reflects our health policy priorities and responds to the needs of the population. One challenge facing health services researchers is developing and sustaining meaningful research priorities and agendas in an overburdened, complex health care system with limited capacity for PHC research and support for clinician researchers.

**Methods:**

A scoping review of the literature was conducted to examine PHC research priorities in Canada. We compared national research priorities for PHC to research priorities being considered in the province of Alberta. Our scoping review was guided by the following questions: (1) What are the research priorities for PHC in Canada?; and (2) What process is used to identity PHC research priorities?

**Results:**

Six key theme areas for consideration in setting a PHC research agenda were identified: research *in* practice, research *on* practice, research *about* practice, methods of priority setting, infrastructure, and the intersection of PHC and population/public health. These thematic areas provide a new framework for guiding PHC research in Canada. It was developed to generate best practices and new knowledge (i.e., innovation), transform PHC clinical practice or support quality improvement (i.e., spread), and lead to large-scale health care system transformation (i.e., scale).

**Conclusions:**

Priority-driven research aims to answer questions of key importance that are likely to have a significant impact on knowledge or practice in the short to medium term. Setting PHC research priorities ensures funded research has the greatest potential population health benefit, that research funding and outputs are aligned with the needs of practitioners and decision makers, and that there is efficient and equitable use of limited resources with less duplication of research effort. Our findings also suggest that a common research priority framework for PHC research in Canada would ensure that research priority-setting exercises are grounded in an evidence-based process.

## Background

Primary health care (PHC) is the foundation of Canada’s health care system [1]. It is where health promotion and chronic disease prevention and management strategies are undertaken, and where patients in need of more specialized services are connected to secondary care. The World Health Organization (WHO) adopted the PHC approach in 1978 as the conceptual basis for effective health care delivery. PHC is an expansive term that takes a holistic and comprehensive view of the factors that contribute to health [2]. According to Mable and Marriott, PHC “recognizes the broader determinants of health and includes coordinating, integrating, and expanding systems and services to provide more population health, sickness prevention, and health promotion. It encourages the best use of all health providers to maximize the potential of all health resources.” (pg. ii) [3].

In Canada, provincial and territorial ministries of health have made significant investments in PHC reform over recent years to improve access, quality and continuity of care, cost, patient satisfaction, and population health outcomes. Interdisciplinary team-based care, networks with streamlined care pathways, health information technologies use, new funding and remuneration models, patient engagement initiatives, chronic disease prevention and management strategies, and new linkages with other key sectors are a few of the many examples of provincial and territorial reforms that have been used in efforts to strengthen PHC in Canada. Despite these investments, the current system remains fragmented and uncoordinated, which causes additional stress, confusion, and potential harm to Canadians in need of care – particularly for those with complex care needs. Scholars have argued that Canada’s stalled commitment to strengthening PHC is a result of poor investment in PHC research and evaluation [4]. To guide our response to these challenges it is critical that research is both driven by and informs health care policies and practice. This goal can be achieved only through robust PHC research that reflects our policy priorities and responds to the needs of the population using diverse research methods.

### The PHC research landscape in Canada

Since the 1980s, Canada’s provinces and territories have, to varying degrees, reformed PHC through small-scale pilot projects that focus on changes in the delivery and organization of PHC services. The largest fund allocated to support PHC transformation in Canada is the Primary Health Care Transition Fund (PHCTF) ($800 M between 2001 and 2006). The PHCTF launched 60 pilot projects across the provinces and territories to support sustainable new approaches to PHC delivery. In addition, a major investment was made to create the Canadian Primary Care Sentinel Surveillance Network (CPCSSN) that receives electronic medical records (EMR) data from primary care clinics across the country, providing a valuable resource for monitoring chronic disease in Canada, as well as for primary health care research. CPCSSN began in 2008 with funding from the Public Health Agency of Canada under a contribution agreement with the College of Family Physicians of Canada and is associated with departments of Family Medicine across Canada. These investments have improved the sustainability of PHC research in Canada. More recently, each province is establishing Support for People and Patient-Oriented Research and Trials (SUPPORT) units that are expected to be multidisciplinary specialized research resource centres. These SUPPORT units are funded through the Canadian Institutes for Health Research, Canada’s Federal funding agency for health research.

One challenge facing health services researchers is developing and sustaining meaningful research priorities and agendas in an overburdened, complex health care system with limited capacity for PHC research and support for clinician researchers. The 2007 report, *Mapping the Future of Primary Health Care Research in Canada,* highlighted deficiencies in the sustainability of Canadian PHC research. For instance, the report outlined the limited capacity for PHC research and support for primary care practitioners and other personnel to be involved in research. PHC researchers have felt isolated in their own organizations or, at least, in their area or region [5]. A 2009 report to the Canadian Health Services Research Foundation (renamed the Canadian Foundation for Healthcare Improvement in 2012) urged Canada to “step up to support and coordinate programs of research about what works in the Canadian context, and enhance the spread and implementation of desired evidence-informed changes.” (pg. 1) [6]. The report argued for the following recommendations to strengthen PHC research in Canada: (1) a coordinating body for PHC research and knowledge transfer; (2) high-level research training and mid-level career support for PHC clinicians and non-clinical research scientists; and (3) the collection and widespread dissemination of PHC related knowledge to practitioners, the community, and policy makers.

These reports concluded that a scattered and uncoordinated mix of individuals and teams were conducting Canadian PHC research. However, this situation is evolving today with the creation of academic positions focused on PHC research [7]. In the past few decades, health services research in general practice/family medicine has drawn on networks of practitioners prepared to participate in research. The evolution of the academic discipline of primary care and growing interest of academic departments of family medicine in PHC research has resulted in more primary care practitioners taking part in research. Academic departments of family medicine and practice-based research networks are contributing to translational research that impacts patient outcomes, reduces costs to the health care system, improves population health, and fosters the work-life balance of PHC practice [7]. The College of Family Physicians of Canada has recently developed a Blueprint for Research Success [8]. The Blueprint calls for all family physicians to be “engaged with research.” This recommendation recognizes the continuum from being a research user (practising evidence-based medicine) on one hand, to devoting the majority of one’s time to research on the other hand. Through capacity building and advocacy the plan seeks to engage academic faculties of medicine, research funders, as well as family physicians in the growth of primary health care research in Canada.

## Review of the literature

We used a scoping review method to outline and identify gaps in the literature related to PHC research priorities in Canada. Scoping reviews are often conducted to examine previous research activity, disseminate findings, identify gaps in the research, and/or determine the value of conducting a full systematic review [9, 10]. Given there are limited comprehensive reviews on PHC research priorities in Canada and the literature is spread across multiple disciplines and sectors, a scoping review method was ideal for taking the first step towards developing a better understanding of the nature and scope of the literature.

## Methods

A scoping review is an increasingly utilized approach for reviewing health research evidence in order to convey the breadth and depth of a field [9]. We conducted a scoping review using an iterative process that allowed for flexibility in the search, reviewing and charting of concepts as recommended by Arksey and O’Malley [9] and Levac, Colquhoun and O’Brien [10]. Scoping reviews involve a process of charting the existing literature or evidence base. We therefore outlined national research priorities for PHC to inform a framework for provincial PHC research priorities in Canada. The results presented in this paper are a review of the academic literature and published reports. We followed Arksey and O’Malley’s [9] framework for conducting scoping reviews including (1) identifying the research question; (2) identifying relevant studies; (3) carefully selecting studies; (4) charting the data; (5) collating, summarizing and reporting the results; and (6) consulting with PHC experts.

### Identification of the research question(s)

Our scoping review was guided by the following review questions:

1. What are the research priorities for PHC in Canada?
2. What process was used to identify PHC research priorities?

### Identification of relevant articles

Our criteria for including relevant papers or sources were: (a) papers or sources that identified priorities for PHC research in Canada; (b) any priority setting exercise that aimed to develop research priorities for PHC in Canada; and (c) health services research. As well, papers or sources that discussed supporting the research infrastructure to facilitate PHC research were included (e.g., investing in research capacity development in PHC) (Table 1). We excluded papers from analysis if the focus was on: (a) clinical effectiveness research priorities; and (b) primary care only and not PHC.

**Table 1:** Types of Literature Reviewed

Literature type	
Commentaries	• E.g., roadmaps for PHC research in Canada or across jurisdictions
Government reports	• Canadian Health Services Research Foundation. A Structure for Co-ordinating Canadian Primary Healthcare Research. February 2009. • Canadian Health Services Research Foundation. Mapping the Future of Primary Healthcare Research in Canada. September 2007. • Canadian Institute for Health Information. Enhancing the Primary Health Care Data Collection Infrastructure in Canada. Report 2. Pan-Canadian Primary Health Care Indicator Development Project. 2006.
Published research	• Published research reporting on the methods for generating research priorities for PHC (e.g., stakeholder meetings, focus groups, Delphi method, priority-setting exercises)
Published research	• Published research on research priority areas for PHC (e.g., chronic disease management, mental illness, breast cancer screening; long-term care)

### Systematic selection of articles

Data collection for the literature review began with searching electronic databases, peer-reviewed academic journals, and reference lists of relevant articles and research documents. For our database searches, we developed a list of search terms and used different combinations of terms to carry out several searches within each database to access the relevant literature. All searches were limited to English language and peer-reviewed papers published in the 10-year period 2005 to 2015. We limited the number of years to make the project feasible and to focus on most recent developments.

We also searched for grey literature using similar search terms in Table 2 in the online databases of the Canadian Foundation for Healthcare improvement (CFHI), Canadian Institutes for Health Information (CIHI), Canadian Institutes for Health Research (CIHR), Canadian Association for Health Services and Policy Research (CAHSPR), Provincial Ministries of Health, and university research centres or institutes. Additionally, we ran a Google Scholar search on the term “research priorities in primary health care” and examined the first 50 pages of the returned results.

**Table 2:** Search strategy for electronic databases

Database	Search Terms
Medline (Ovid)	“research priorities” “primary health care” “Canada”
PubMed	priorities [All Fields] AND (“primary health care” [MeSH Terms] OR “primary health care” [All Fields]) AND (“research” [MeSH Terms] OR “research” [All Fields]) AND Canada
CINAHL	(research priorities) AND (primary health care) AND Canada

### Charting the data

In total we identified 672 abstracts, which we imported into Endnote™, a reference management software, to facilitate initial screening of abstracts and titles. Articles that were not focused on research priority setting for PHC in Canada were initially removed (*n* = 212). Duplicates and irrelevant titles were also removed. We retained 25 articles that closely met our inclusion criteria for analysis. Each of the 25 articles was read thoroughly and all pertinent information was extracted including the jurisdiction in which the study was conducted, type of article (empirical conceptual, or grey literature), method of research priority-setting used (if applicable), and key messages on PHC research. This information was then entered onto a charting form using Microsoft Excel by the lead author (SM) and double-checked by a second co-author (ARV).

### Consultation with experts

We consulted with a small number of leading PHC research experts in Canada about our initial search findings, recommendations for additional sources to include in our review that may have been missed in our search strategy, and their opinion of research gaps in PHC.

## Results

We identified six key theme areas that emerged as priorities for Canadian PHC research. The first three themes were categorized as (1) generate best practices and new knowledge, (2) transform PHC clinical practice or support quality improvement, and (3) lead to large-scale health care system transformation. Additionally, we found (4) information about the methods preferred or most often used for PHC research and for setting PHC research priorities, (5) the imperative for supportive research infrastructure, and (6) the opportunity to intersect with public/population health for a deeper inclusive understanding of the most urgent health needs facing Canadians. In our review we did not find a robust framework for guiding provincial PHC research agendas. In the following section we describe in more detail these themes, which create a framework for PHC research priorities.

## Key themes

### Research in practice

Research *in* practice is the creation of practice-based evidence whereby research questions are generated by practising family physicians and PHC teams. Practice-based research networks offer a model where general practices form the natural laboratories wherein research is undertaken and knowledge translated into practice. Networks come in different forms, corresponding to different purposes and functions, for example, (1) sentinel surveillance networks for the early identification of threats to public health [11]; (2) quality assurance networks focused on changing the behaviour of providers; (3) networks for clinical trials (e.g., pragmatic clinical trials and double-blind randomized controlled trials); and (4) intervention networks to provide information and rapid turnaround about specific interventions [12]. There is growing evidence of the value of practice-based research networks for linking research to practice improvement and speeding the dissemination of research findings to community practice settings [11, 13]. However, funding for network activities, infrastructure, and operations is described as a barrier to realizing the potential of practice-based research networks as a research resource [13].

### Research on practice

Research *on* practice includes research that studies how to do the best job of what we do (i.e., quality improvement), such as how to implement new models of care delivery and new organizational structures that have demonstrated lower costs and improved health outcomes. As an example, one study in our literature review focused on practice-level and organizational change in the delivery of equity-oriented PHC services for marginalized populations [14]. While there is growing research on health equity and the social determinants of health, research is needed to assess how Canada is performing with respect to heath equity, both within and across provinces/territories. Equity-focused PHC research will inform decision makers on how to reorient the health care sector as one that makes health equity a central goal and, in so doing, engages with the entire range of social determinants of health.

### Research about practice

Research *about* practice is research that creates a paradigm shift in the PHC system, particularly around the organization and delivery of PHC services, how services are paid for, and changes that promotes the long-term sustainability of the health care system as a whole. For example, Ashbury and colleagues identified research priorities for a prevention intervention research agenda aimed at scalable recommendations to achieve improved population health [12]. Also important to PHC transformation in Canada is promoting effectiveness of interprofessional care teams. Jones and Way explained that while the transition to team-based care has been embedded in PHC reforms across Canada, systematic comparisons of interprofessional teams across diverse settings are limited [15].

The need for a comprehensive PHC population-based data collection strategy to assess specific qualities of PHC services (e.g., coordination and effectiveness) and most immediate outcomes of PHC was identified as a research and policy priority in Canada [1, 16]. A PHC data collection strategy and information system is needed to inform policy development and planning, to evaluate PHC initiatives, and to meet the expectations of Canadians regarding health care system accountability [16].

### Methods of priority setting

Interdisciplinary work and the integration of rigorous methods from the range of quantitative and qualitative disciplines are needed to address PHC research questions [17]. Collaborations in PHC research among researchers, the public, practitioners, and policy makers are also necessary to have an impact on community practice or demonstrate a benefit at a population or local level [5]. In the reviewed literature, we found five specific methods for research priority setting: (1) strategic planning workshop; (2) symposium; (3) Delphi method; (4) agenda setting meeting; and (5) stakeholder dialogue.

### Infrastructure

Widespread reform of the PHC sector in Canada and the increasing importance of PHC to health outcomes have created a need to build research capacity in this sub-discipline of health care and health services research. The best way of determining the value for money spent in PHC reform is through research that studies the effects of increased (or decreased) funding, comparing results from jurisdiction to jurisdiction and examining the data on patient outcomes. We found eleven studies that described the organization, process, and resources needed for PHC research in Canada. Several infrastructure supports have been put in place to accelerate PHC research across Canada [1]. For instance, in 2008 the Canadian Health Services Research Foundation formed the Canadian Working Group for Primary Health Care Improvement (CWGPHI) to promote evidence-informed PHC practice and policy. A primary objective of the working group was to establish a common set of indicators for PHC performance [1]. PHC researchers, as members of the working group, were encouraged develop indicators that would be meaningful in various contexts and useful in national and international comparisons.

Ontario was referenced in the literature as employing a “pump-priming” model organized around building research capacity and facilitating research transfer through infrastructure grants and career awards toward making their provincial health researchers – and research institutions – more competitive in the CIHR competitions. Few health care research centres focused on PHC scholarship exist in other provinces. Furthermore, limited research capacity in PHC in Canada is described in the literature as an impediment to strengthening the PHC system in Canada [18, 19]. In 2003, the Canadian Institutes for Health Research established a national strategic training program, the Transdisciplinary Understanding and Training on Research in Primary Health Care (TUTOR-PHC), at Western University in London, Ontario in order build a critical mass of skilled and independent researchers through student opportunities and faculty development; and increase the interdisciplinary focus in PHC research. Given the importance of interdisciplinarity in PHC research, the program provides a valuable opportunity for clinical and non-clinical researchers across the province to consolidate their interests and identify priorities for PHC research [19]. However, continued investment in capacity-building programs and retaining world-class PHC researchers in Canada remains a challenge [19].

Scholars have also reported on the institutional challenges with conducting pan-Canadian health surveillance research that is essential to informing PHC policy and practice [11]. For instance, Kotecha and colleagues reported that ethics review boards and privacy concerns are barriers for practice-based disease surveillance systems in Canada such as CPCSSN [11], which collects and validates longitudinal PHC information (e.g., hypertension, diabetes, depression, chronic obstructive lung disease, osteoarthritis). Researchers face challenges in obtaining consistent Institutional Research Ethics Board (IREB) approvals, and a lack of clarity in guidelines has impeded advances in Canadian health surveillance and research.

### Intersection of primary health care and public/population health

The reviewed literature emphasized the importance of collaboration between the PHC and public health sectors to strengthen the Canadian health care system. A Health Canada report from a Canadian Public Health and Primary Health Care Workshop stated that, since examples of successful collaborations between primary care (PC) and public health (PH) exist, future research was needed to document what has worked and what lessons have been learned [20]. Valaitis and colleagues addressed this gap by exploring the structures and processes required to build successful collaborations between PC and PH to understand the nature of existing collaborations in Canada [21]. They also examined the roles that nurses and other providers played in collaborations. Using social ecological theory to examine collaboration between PC and PH, they found that determinants of collaboration at one level enhance or suppress contextual factors that influence collaboration at another level (i.e., system, organizational, interpersonal, and intrapersonal levels). They argue that future research needs to focus on identifying indicators of successful collaborations as well as measuring process and outcomes of collaborations.

## Discussion

Six key theme areas for consideration in setting a PHC research agenda for Canada were identified through the scoping literature review: research *in* practice, research *on* practice, research *about* practice, methods of priority-setting, infrastructure, and the intersection of PHC and population/public health. These thematic areas provide a new framework for guiding PHC research in Canada (Table 3).

**Table 3:** Framework for PHC research priorities

PHC Research Priorities	Description
Research *in* practice	Action research. Creation of practice-based evidence that works in the real world to benefit the patients of participating physicians/clinics. Innovation.
Research *on* practice	Implementation science; quality improvement. Directly useful to the specific physicians/clinics that participate in the research. Practice/clinical transformation. Spread.
Research *about* practice	Knowledge-generating research that is not specific to a particular physician/clinic but is more generalized to the population as a whole and to the health care system (e.g., policy research, theory-building research, system transformation). Scale.
Methods	Research methods that are the most appropriate (or most often used) in PHC research. Promising new approaches.
Infrastructure	Organizations, processes, and resources needed for PHC research in Canada.
Intersection of PHC and public/population health	The need for population health data for priority setting and collaborations to put knowledge into action.

The scoping review highlighted the importance of practice-based research and the role of networks in performing research *in* practice, but no authors suggested a research agenda for studying practice-based research networks and the impact these networks have on PHC in Canada. Nevertheless, the U.S. experience with practice-based research networks has demonstrated that research that is grounded in and informed by PHC providers is important to identifying common problems in clinical contexts, demonstrating the effectiveness of treatments in real-world settings, and determining how to maximize the benefits of improvements in PHC delivery for patients [22]. Operating at the intersection of research and quality improvement, practice-based research networks focus on practice-relevant research questions defined by participating PHC providers, apply appropriate multi-method research designs, and include providers in the interpretation of results. Practice-based research networks are not only rich laboratories for describing patient-provider encounters, they serve also as means for introducing innovations in practice that, in theory at least, will lead to improvements in population health status. Research in this area is currently underway in Canada. A recent CIHR funded project is carrying out a provincial project among British Columbia, Ontario, and Quebec to demonstrate the feasibility and usefulness of a comparative and comprehensive community-based PHC performance measurement and reporting in the three provinces, as a foundation to inform innovation in the delivery and organization of the Canadian community-based PHC system [23].

Our findings from the literature review do not describe the challenges with collecting data from electronic medical records (EMR), other than articulating the barriers to research imposed by ethics review boards and privacy legislation in the provinces. In some primary care settings, groups of providers have their own analysts that have created dashboards to extract data from electronic medical records (EMR) in order to assess adherence to clinical practice guidelines (e.g., Choosing Wisely Canada) and comparing provider practice indicators (e.g., time to next appointment). There is, however, no way to determine patients’ use of secondary level services (e.g., Emergency Departments, Urgent Care Centres) because there is no connection between EMR data from providers and administrative data from acute care.

Practice-based research networks are also conduits for research *on* practice – research that helps to spread innovations from pilot projects in small clinics to larger practice networks to foster PHC practice transformation. In this, there is an important role for health services researchers in collaborations among academic institutions, community-based PHC teams, and patients to address questions about how PHC services are organized, delivered, and evaluated as well as how knowledge about best practices is disseminated. For example, various models of patient attachment, panel management, team-based care, and clinical pathways and guidelines application can be compared in different settings to ascertain the most effective models. Our findings suggest that there is limited understanding of how PHC providers change their practice to adopt innovations and of the barriers and facilitators for altering PHC service delivery to accommodate new knowledge. Further, the growing interest in patient-engaged research offers opportunity to apply another lens to research on PHC practice and the patient experience.

The Diffusion of Innovation Theory [24] helps to explain how, why, and at what rate new ideas spread. Diffusion is “the process by which an innovation is communicated over time among participants in a social system.” [24] Rogers categorizes individuals into five groups on the basis of the time they begin using a new idea: (1) Innovators; (2) Early adopters; (3) Early majority; (4) Late majority; and (5) Laggards [24]. Current research is underway in the province of Alberta to understand the differences in mental models between early adopter and early majority physicians regarding primary care transformation on the medical home model. “Mental model” is the term cognitive and organizational scientists apply to an individual’s or team’s understanding of how some aspect of the world around them does and should work. In PHC this would include the clinical work of providers or care teams, what their roles are, and how care delivery processes work.

Research *about* practice, therefore, is research that focuses on system transformation; that is, the scale up of innovations and best practices. Key factors that influence the success of scale-up efforts include the features of innovations, the target settings, the role of champions in promoting implementation, and the effective strategies for facilitating the scale-up and spread of innovations [25]. For example, the adoption of population-based chronic disease management in PHC practice settings has supported change in the health care system from one that is designed for episodic, acute illness to one that will support the prevention and management of chronic disease [26]. Our review highlighted the association between prevention intervention research [26], quality improvement research, and system transformation.

Research priority setting processes assist PHC researchers and policymakers in effectively prioritising research that has the greatest potential population health benefit. Many different approaches to research priority-setting were identified in the scoping review, but there is no consensus on best practice for PHC research prioritisation. Moreover, patient involvement in research priority-setting exercises was not described. Engaging patients, caregivers, and health care providers in establishing research priorities ensures that resources are invested to answer questions that address the shared priorities in PHC research.

While the literature reviewed suggested there is a need for improved collaboration between primary care and public/population health sectors, this research priority is neither simple nor natural to achieve. There is, for example, little overlap in the training of PHC practitioners in population health or public health. Without a clear understanding of the contexts of practice, there is little opportunity to take advantage of the potential for intersectional research. There is a paucity of health services researchers, making it difficult to conduct this type of research to the level required to transform practice.

## Conclusions

Priority-driven research aims to answer questions of key importance that are likely to have a significant impact on knowledge or practice in the short to medium term. Setting PHC research priorities ensures that funders support research that has the greatest potential population health benefit, that research dollars and outputs are aligned with the interests of practitioners and decision makers, and that there is less duplication and more efficient and equitable use of limited resources.

Our findings recognized particular issues associated with research in the PHC setting, such as its contextual complexity. Future directions should support an infrastructure for PHC research capacity by continuing to offer training opportunities for academic researchers and PHC practitioners engaged in clinical research. Moreover, PHC research should be grounded in the community in order to generate appropriate and equity-focused health evidence for health care teams, patients, and policy makers. In turn, academic institutions need to be more responsive to community-based research efforts that are more complex than research conducted in (for example) acute care settings.

Our findings also suggest that a common research priority framework for PHC research in Canada would ensure that research priority-setting exercises are grounded in evidence-based processes. Our framework for PHC research priorities is a promising way forward to promote the value of frameworks as knowledge translation mechanisms to guide a PHC research agenda. A further stage of priority setting is now required, drawing on the research community’s specific expertise and experience and patient and public engagement in the identified thematic areas, to assess gaps in research that is relevant to local, regional and provincial PHC delivery in Canada.

## Abbreviations

CIHR: Canadian Institutes for Health Research
CPCSSN: Canadian Primary Care Sentinel Surveillance Network
PHC: Primary health care
SUPPORT: Support for People and Patient-Oriented Research and Trials
